# Patient preferences for characteristics of antiretroviral therapies: results from five European countries

**DOI:** 10.7448/IAS.17.4.19540

**Published:** 2014-11-02

**Authors:** Brian Gazzard, Shehzad Ali, Axel Muhlbacher, Neda Ghafouri, Franco Maggiolo, Catherine Golics, Silvia Nozza, Maria Jose Fuster, Antonio Antela, Jean Jacques Parienti, Nathalie Dang, Sylvie Ronot Bregigeon, Andrew Benzie, Miranda Murray

**Affiliations:** 1 HIV and Sexual Health, Chelsea & Westminster, London, UK; 2 Health Economics, ICON plc, Oxford, UK; 3 Economics, IGM Institute of Management, Neubrandenburg, Germany; 4 Medical Affairs, ViiV Healthcare, Munich, Germany; 5 Antiviral Therapy Unit, Bergamo, Italy; 6 Patient Reported Outcomes, ICON plc, Oxford, UK; 7 Vaccine and Immunotherapy Research, IRCCS, San Raffaele, Italy; 8 SEISIDA, Madrid, Spain; 9 H. Conxo, S de Compostela, Spain; 10 Biostatistics and Clinical Research, Faculte de Medecine de Caen, Caen, France; 11 Medical Affairs, ViiV Healthcare, Singapore, Singapore; 12 Immunology and Hematology, CHU Sainte – Marguerite, Marseille, France; 13 Medical Affairs, ViiV Healthcare, London, UK; 14 Health Outcomes, ViiV Healthcare, London, UK

## Abstract

**Introduction:**

Patient preference to antiretroviral therapy (ART) characteristics should be a key consideration in treatment decisions. ART options exist for people living with HIV (PLWH), however concerns remain related to PLWH satisfaction with current ARTs. The current study examines patient preferences and the strength of preferences for treatment characteristics associated with ART.

**Materials and Methods:**

Patients’ preferences to ART were explored using a discrete choice experiment (DCE). Seven defined treatment characteristics (each with three categories) were identified from a literature review, input from experts, PLWH and physicians. A total of 1582 PLWH from France, Germany, Spain, Italy and the UK were recruited for the study. An adjusted odds ratio <1 signified lower odds of selecting a treatment with this characteristic category, compared to the reference category, independently of other characteristics.

**Results:**

The patient preference analyses showed that participants preferred treatments with a rapid reduction in viral load (OR=0.78; 95% CI 0.74–0.81) and CD4 count (OR=0.86; 95% CI=0.82–0.89). Participants had a strong preference for avoiding diarrhoea (Odds ratio, OR=0.36 95% CI=0.33–0.38) and long term health problems (OR=0.30, 95% CI=0.28–0.32). Convenience related issues related to restrictions on taking drugs because of food or drug interactions were important to avoid (OR=0.80, 95% CI=0.76–0.83 and OR=0.72 95% CI=0.69–0.76 respectively). Participants also had a strong preference to avoid drugs which limited the effectiveness of future treatments (OR=0.70, 95% CI=0.67–0.73).

**Conclusions:**

Avoidance of diarrhoea and long-term complications were the most important drivers of patient choice. This study, from a large sample of European patients, demonstrates the importance to patients when different aspects of HIV treatment are considered simultaneously.

**Table 1:** Results

Variables (reference category)	Odds ratio* (95% Confidence Interval)
**Viral load (reference: undetectable)**	
400 copies/mL in 4 weeks, undetectable after 3 months	0.87 (0.83–0.91)
1000 copies/mL in 4 weeks, undetectable after 3 months	0.78 (0.74–0.81)
**CD4 cell count (reference: increase of CD4 + 100 m^3^)**	
Increase of CD4 +50/mm^3^ after 3 months	0.91 (0.87–0.95)
Increase of CD4 +25/mm^3^ after 3 months	0.86 (0.82–0.89)
**Diarrhoea (reference: no diarrhoea)**	
3 episodes of diarrhoea/day	0.71 (0.68–0.74)
> 6 episodes of diarrhoea/day	0.36 (0.33–0.38)
**Long-term health problems (reference: no increased risk)**	
10% risk of future health problems	0.55 (0.53–0.57)
20% risk of future health problems	0.30 (0.28–0.32)
**Treatment failure (reference: all ARTs available)**	
ARTs only partially effective	0.79 (0.75–0.82)
Some ARTs can't be used; others only partially effective	0.70 (0.67–0.73)
**Food restrictions (reference: no food requirements)**	
ARTs with food	0.93 (0.89–0.97)
ARTs on empty stomach	0.80 (0.76–0.83)
**Drug-drug interactions (reference: no drug interactions)**	
ART dosage adjusted; may increase risk of side effects	0.75 (0.72–0.79)
Cannot take certain medications	0.72 (0.69–0.76)

* The results indicated significant p-values<0.05 for each variable.

